# Blood pressure and hypertension prevalence among oldest-old in China for 16 year: based on CLHLS

**DOI:** 10.1186/s12877-019-1262-4

**Published:** 2019-09-09

**Authors:** Jiajun Du, Guoning Zhu, Yanhong Yue, Miao Liu, Yao He

**Affiliations:** 1 Medical Information Center, Chinese PLA General Hospital, Beijing, China; 2 Oncology Department of the Second Medical Center, Chinese PLA general hospital, Beijing, China; 3 Medical Service, National Defense Mobilization Department, China Military Commission, Beijing, China; 4 Beijing Key Laboratory of Aging and Geriatrics, National Clinical Research Center for Geriatrics Diseases, Institute of Geriatrics, State Key Laboratory of Kidney Diseases, Second Medical Center of Chinese PLA General Hospital, Beijing, China

**Keywords:** Blood pressure, Hypertension, Prevalence, Oldest-old, Epidemiology

## Abstract

**Background:**

There were little national data on hypertension based on the oldest-old, and lack of information on chronological changes. This study aimed to describe trends of blood pressure (BP) levels and hypertension prevalence for the past 16 years among the oldest-old in China.

**Methods:**

All the oldest-old who had participated in the Chinese Longitudinal Healthy Longevity Survey (CLHLS) 1998 to 2014 with information about BP levels and hypertension were included in the analysis.

**Results:**

There was fluctuation over the past 16 years for both SBP and DBP levels. The mean SBP level decreased from 148.4 ± 24.4 mmHg in 1998 to 130.8 ± 18.7 mmHg in 2005, and then increased to 139.7 ± 22.0 mmHg in 2014. The mean DBP level decreased from 84.3 ± 13.4 mmHg in 1998 to 78.9 ± 11.7 mmHg in 2008, and then increased to 79.7 ± 11.8 mmHg in 2014. The hypertension prevalence increased from 43.1 to 56.5% for the 16 years. The prevalence of isolated systolic hypertension was lowest in 2002–2005 (14.3%), and then increased to 30.7% in 2014. Multivariate logistic regression showed that older age, lower education and economic level, without health insurance were associated with higher hypertension prevalence.

**Conclusions:**

There was a significant increase in hypertension prevalence among the Chinese oldest-old from 1998 to 2014. Greater efforts are needed for hypertension prevention among this specific population.

## Background

Hypertension is one of the important risk factors for cardiovascular disease. The higher the blood pressure (BP), the greater the coronary heart disease and stroke risks [1–3]. Therefore, it is particularly important to understand the epidemic trend of hypertension. The prevalence of hypertension varies greatly among different age groups, especially among elderly. And isolated systolic hypertension (ISH) (systolic blood pressure (SBP) ≥140 mmHg while diastolic blood pressure (DBP) < 90 mmHg) was most existed in elderly [4, 5].

On the other hand, there was a lack of basic data about hypertension prevalence among the oldest-old (aged 80 and over) in China. Previous studies were either among adults, or with small sample, or mainly with inpatients [6, 7]. There were little national data on hypertension based on the oldest-old. Additionally, most studies were based on one time survey, the chronological changes had never been reported, which was a reflection of effects about national control measures of hypertension.

Therefore, we reported the epidemiology characteristics of hypertension based on 63 thousand oldest-old from seven waves (1998, 2002, 2002, 2005, 2008, 2011, 2014) of Chinese Longitudinal Healthy Longevity Survey (CLHLS), the first and largest longitudinal survey focused on the oldest-old in China [8]. We evaluated the prevalence of hypertension by geography and subpopulations, and the chronological changes.

## Methods

### Study design

All the participants were from the seven waves of CLHLS, and those who aged more than 80 years old with complete records on BP and hypertension information were included. General characteristic of the seven survey waves was listed in Table 3 in Appendix. The details of the CLHLS and sample design have been described elsewhere [8]. The follow-up survey waves were conducted in 2000, 2005, 2008, 2011, and 2014. The use of CLHLS data was approved by the Biomedical Ethics Committee of Peking University, and written informed consent was obtained from each respondent.

### Definitions

According to the BP levels, participants were divided into the following groups: normal BP, SBP ≤ 120 mmHg and DBP ≤80 mmHg among those who had never been diagnosed with hypertension; high-normal BP, 120 mmHg<SBP ≤ 139 mmHg or 80 mmHg<DBP ≤ 89 mmHg among those who had never been diagnosed with hypertension; Hypertension, SBP ≥ 140 mmHg or DBP ≥ 90 mmHg or self-reported being diagnosed as hypertension by II&III grade hospital before; ISH was defined as SBP ≥ 140 mmHg and DBP < 90 mmHg regardless of previous hypertension diagnosis history. Mean arterial pressure (MAP) was calculated as the following formula: (SBP+ (2 × DBP)) ÷3. Pulse pressure (PP) was calculated as SBP minus DBP.

### Statistical analysis

Mean SBP, DBP, MAP, and PP levels were calculated and expressed as mean ± standard deviation (SD). Variance analysis was used to compare the differences among subgroups. Besides, we estimated the prevalence of hypertension among all the participants. The age and gender adjusted prevalence of hypertension for the first four waves (1998, 2000, 2002, 2005) was calculated using the direct methods based on the fifth Chinese national census data, and the age and gender adjusted prevalence of hypertension for the last three waves (2008, 2011, 2014) was calculated using the direct methods based on the fifth Chinese national census data. Multivariate logistic regression was used to calculate Odds ratios (ORs) and their 95% confidence intervals (CIs).

### Ethical consideration

The use of CLHLS data was approved by the Biomedical Ethics Committee of Peking University.

## Results

### Trends of BP levels

**Fig. 1:**
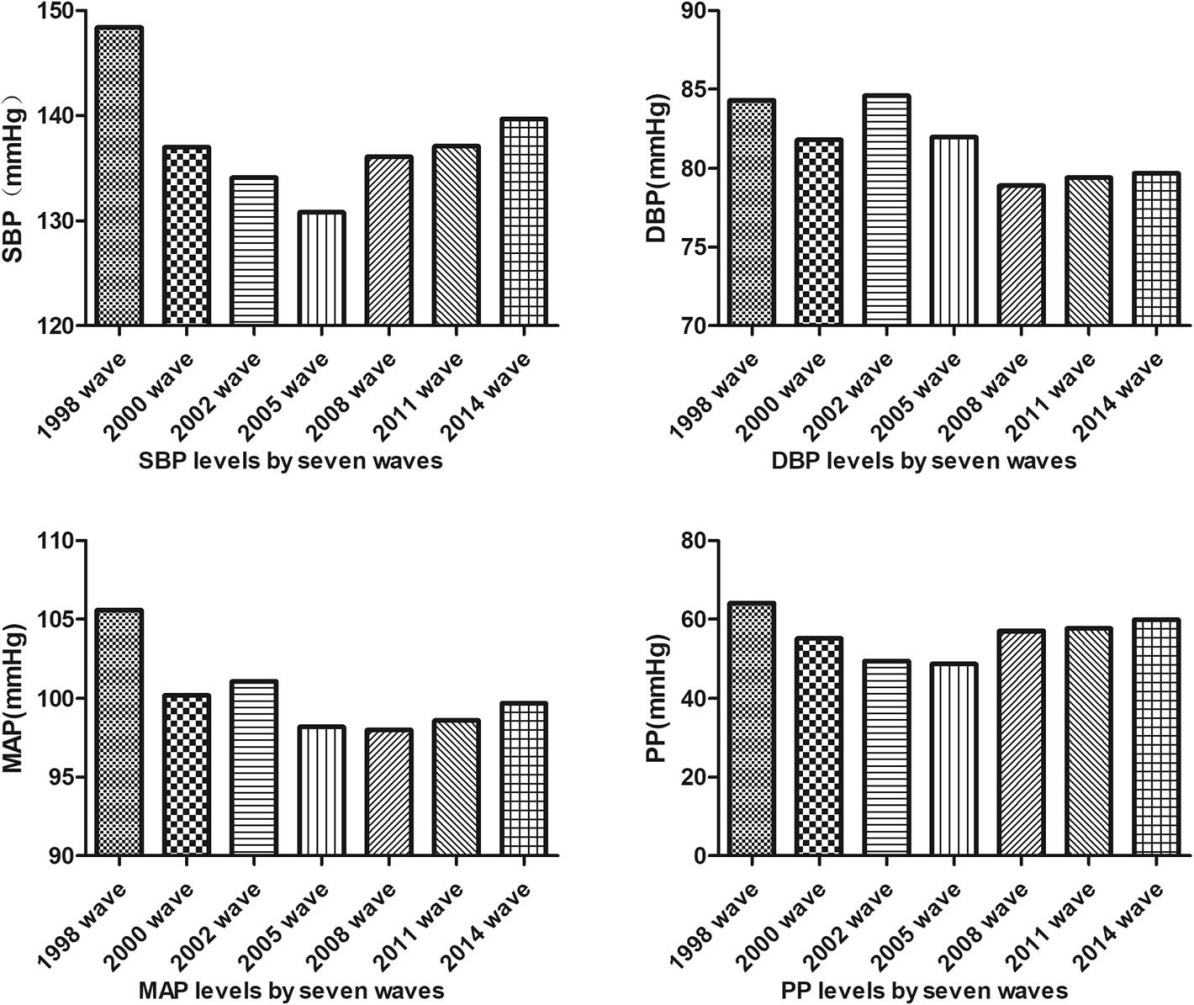
SBP, DBP MAP and PP levels for the seven survey waves

Figure 1 showed the trends of BP levels among the seven waves. The mean SBP level decreased from 148.4 ± 24.4 mmHg in 1998 to 130.8 ± 18.7 mmHg in 2005, and then increased to 139.7 ± 22.0 mmHg in 2014. There was fluctuation over the past 16 years for SBP levels. For different age groups, those aged 80–89 years had the highest SBP levels, while those aged ≥100 years had the lowest SBP levels(p < 0.05). Compared with different age groups, SBP level was the highest in 80-year-olds and lowest in 100-year-olds (p _for trend_ < 0.05). For different categories of residence, those from rural areas had the highest SBP levels, while those from the city had the lowest SBP levels. This trend was pronounced after 2008 wave (p < 0.05). There was no significant difference for male and female (*p* > 0.05). When we excluded those who had hypertension, the trend was similar to that of total population (Table 4 in Appendix). The mean DBP level decreased from 84.3 ± 13.4 mmHg in 1998 to 78.9 ± 11.7 mmHg in 2008, and then increased to 79.7 ± 11.8 mmHg in 2014. Data showed that there had been fluctuations in the seven survey waves during the 16 years (Table 5 in Appendix). The mean MAP level was 105.6 ± 15.2 mmHg in 1998 wave. And it showed decreasing trend until 2008 survey wave (the lowest mean MAP level was 98.0 ± 12.3 mmHg). Then it went up to 99.7 ± 13.3 mmHg in 2014 wave (Table 6 in Appendix). The mean PP level was 64.1 ± 19.8 mmHg in 1998 wave. And it showed decreasing trend until 2005 survey wave. Then it went up to 59.9 ± 18.8 mmHg in 2014 wave (Table 7 in Appendix). There was no significant gender difference for DBP, MAP or PP levels, just like SBP levels. The differences among age, category of residence were similar with SBP levels.

### Trends in hypertension prevalence

In 1998 wave, 43.1% (95%CI: 42.0–44.1%) of the participants had hypertension (Table 1). And the prevalence showed an increasing trend along with seven survey waves over the past 16 years (p < 0.001). The weighted prevalence (two weighted method: 1. weight calculated based on age-sex-residence-specific distribution from the CLHLS study; 2. weight was calculated based on the fifth (for the first four waves, 1998, 2002, 2002, 2005) and sixth (for the last three waves, 2008, 2011, 2014) national census data.) was similar like unadjusted initial value, with the same trend. Similar to BP levels, there was also a significant decrease trend along with age groups (p < 0.05), relatively higher prevalence in rural areas and eastern China (*p* < 0.05), and no significant difference for male and female (*p* > 0.05).

**Table 1:** The prevalence (%) of hypertension by seven waves

Wave	1998	2000	2002	2005	2008	2011	2014	p
Gender								
Male	43.1(41.4–44.7)	43.3(41.9–44.8)	42.3(40.9–43.9)	48.8(47.3–50.3)	46.3(44.8–47.8)	50.1(48.1–52.1)	55.3(53.0–57.5)	< 0.001
Female	43.1(41.7–44.4)	43.8(42.6–45.1)	43.4(42.3–44.6)	46.4(45.2–47.6)	45.8(44.6–47.0)	54.6(53.0–56.2)	57.4(55.5–59.3)	< 0.001
p	0.995	0.607	0.277	0.016	0.602	< 0.001	0.150	
Age-group								
80–89 yrs	45.4(43.7–47.1)	44.3(42.9–45.7)	44.5(43.0–46.0)	51.3(49.8–52.9)	50.2(48.6–57.1)	56.7(54.8–58.6)	59.9(57.9–62.0)	< 0.001
90–99 yrs	43.9(42.1–45.7)	44.3(42.7–45.9)	43.5(42.7–45.9)	46.9(45.4–48.5)	46.4(44.9–47.9)	52.6(50.6–46.6)	54.3(51.8–56.7)	< 0.001
100- yrs	38.7(36.7–40.6)	41.2(39.2–43.2)	40.5(38.8–42.3)	42.3(40.4–44.1)	39.9(38.8–42.3)	46.0(43.4–48.6)	52.2(48.9–55.6)	< 0.001
p _for trend_	< 0.001	0.032	0.003	< 0.001	< 0.001	< 0.001	< 0.001	
Category of residence								
City	41.8(40.1–43.5)	43.2(41.7–45.1)	43.0(41.1–44.9)	45.9(44.6–47.2)	43.7(41.7–45.8)	48.8(43.8–51.7)	51.1(41.2–55.1)	< 0.001
Town		43.4(41.5–44.9)	43.0(41.3–45.1)	48.4(46.5–50.3)	46.1(44.9–47.3)	51.0(48.8–53.3)	56.2(53.7–58.8)	< 0.001
Rural	43.8(42.3–45.3)	44.2(41.7–46.3)	43.2(41.7–44.3)	50.0(47.8–52.0)	48.9(46.9–51.0)	55.1(53.4–56.8)	58.0(56.1–59.9)	< 0.001
p _for trend_	0.123	< 0.001	0.634	< 0.001	< 0.001	0.006	0.838	
Total	43.1(42.0–44.1)	43.6(42.7–44.5)	43.0(42.1–43.9)	47.3(46.4–48.3)	46.0(45.1–46.9)	52.8(51.6–54.0)	56.5(55.1–58.0)	< 0.001
Weighted Total^†^	45.2(44.2–46.3)	44.8(43.9–45.8)	44.4(42.4–46.4)	50.6(48.6–52.6)	50.2(48.2–52.2)	53.8(51.6–55.9)	57.4(55.1–59.7)	< 0.001
Weighted Total^†^	45.3(43.8–46.9)	44.3(43.1–45.6)	44.6(43.2–45.9)	50.9(49.5–52.3)	49.8(48.5–51.3)	56.6(54.8–58.3)	59.5(57.6–61.4)	< 0.001

*:City and town were combined as one category in 1998 wave

^†^: Weight was calculated based on age-sex-residence-specific distribution from the CLHLS study

^‡^: Weight was calculated based on the sixth national census data

The trend for ISH prevalence was different. The lowest prevalence was in 2002 wave (14.3, 95%CI: 13.7–15.0%), the highest prevalence was 30.5% (95%CI: 29.6–31.5%) in 1998 wave and 30.7% (95%CI: 29.3–32.0%) in 2014 wave. The differences among gender, age, category of residence were similar to that of BP levels (Table 8 in Appendix).

**Fig. 2:**
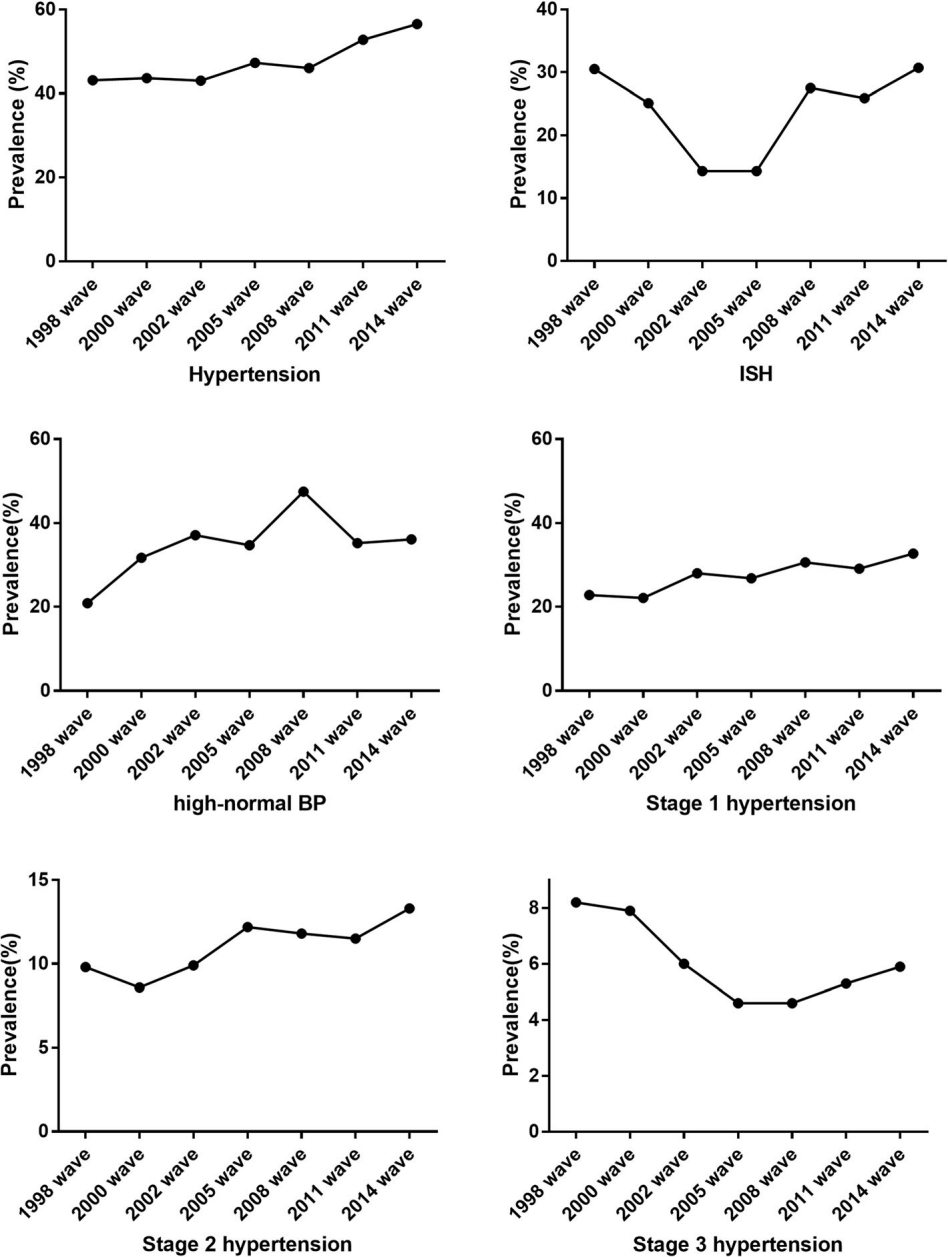
Prevalence of hypertension for the seven survey waves

The prevalence of high-normal BP also showed an increasing trend. It went from 20.9% (95%: 20.0–21.7%) in 1998 wave to the highest of 47.5% (95%: 46.6–48.4%) in 2008 wave. Then it remained at about 35.0% in the following two waves. For different stages of hypertension, participants with hypertension who were classified as stage I or stage II also showed similar increasing trend (Fig. 2).

Sensitivity analysis was performed among those who were first included in the analysis. The BP levels, the prevalence of hypertension were a little higher than the results of the total participants in each survey wave (Table 9 in Appendix).

### Multivariable analysis of hypertension prevalence

Using the most current survey wave data (CLHLS 2014), Table 2 presented the adjusted ORs and 95%CI for the association between covariates in four multivariable logistic regressions each using the following two binary outcomes as dependent variables: (1) prevalence of hypertension; (2) prevalence of ISH. Gender, marriage status had no statistical association with hypertension prevalence. Compared with Han nationality, minorities had lower hypertension prevalence (OR = 0.76, 95%CI: 0.59–0.99). However, only participants aged 90–99 and ≥ 100 years old were of lower risk of hypertension than those aged 80–89 years old (OR = 0.69, 95%CI:0.58–0.79; OR = 0.52, 95%CI: 0.42–0.64 respectively). Participant who had higher education levels, central obesity, were more likely to have hypertension. For different categories of residence, compared with those from urban (including city and town) areas, those from rural areas had higher risk of prevalence of hypertension (OR = 1.24, 95%CI: 1.08–0.99; OR = 0.53, 95%CI: 0.35–0.82 respectively).

**Table 2:** Adjusted ORs (95%CI) for prevalence, of hypertension

Characteristic	Prevalence	Prevalence of isolated systolic hypertension
Gender		
Male	1.00(ref)	1.00(ref)
Female	1.03(0.82–1.29)	0.96(0.80–1.14)
Age-group		
80–89 yrs	1.00(ref)	1.00(ref)
90–99 yrs	0.69(0.58–0.79) *	0.99(0.86–1.15)
100- yrs	0.52(0.42–0.64) *	0.98(0.82–1.18)
Education years		
0 years	1.00(ref)	1.00(ref)
1–6 years	1.06(0.89–1.27)	1.03(0.87–1.22)
≥ 7 years	1.19(0.79–1.63)	0.87(0.65–1.17)
Current marriage		
Married	1.00(ref)	1.00(ref)
Divorced/Widowhood/other	1.10(0.93–1.30)	1.05(0.89–1.23)
Nationality		
Han	1.00(ref)	1.00(ref)
Minority	0.76(0.56–0.99) *	0.86(0.66–1.12)
Smoking		
Never smoking	1.00(ref)	1.00(ref)
Ever smoking	1.36(1.08–1.70)*	0.93(0.75–1.16)
Current smoking	1.03(0.82–1.30)	0.92(0.74–1.14)
Alcohol drinking		
Never drinking	1.00(ref)	1.00(ref)
Ever drinking	1.21(0.96–1.54)	0.89(0.70–1.13)
Current drinking	0.81(0.66–1.02)	0.88(0.71–1.09)
Central obesity		
No	1.00(ref)	1.00(ref)
Yes	1.76(1.52–2.03)*	1.46(1.27–1.68)*
Category of residence		
City	1.00(ref)	1.00(ref)
Town	1.13(1.09–1.34)*	1.21(0.98–1.51)
Rural	1.24(1.08–1.47)*	1.24(1.01–1.53)*
Having health insurance		
No	1.00(ref)	1.00(ref)
Yes	1.05(0.88–1.24)	0.86(0.74–1.01)

*:*p* < 0.05

## Discussion

Our study provided the mean BP levels and epidemic picture of hypertension among the oldest-old in China based on CLHLS 1998 to 2014 survey wave. The results indicated that BP levels were high especially DBP and PP levels. Besides, hypertension was of relatively high prevalence, and showed an increasing trend along with seven survey waves over the past 16 years.

The study of BP levels had filled in the blanks for mean BP levels based on community oldest-old in China. And there was fluctuation over the past 16 years for both SBP and DBP levels.. The mean BP levels were significantly higher than those of adults while they were about the same as those of the younger elderly who aged 65–74 years old from the interASIA study in 2000–2001 [9, 10]. The above results and the comparison with the domestic and foreign studies indicated that the BP levels of the oldest-old in China was close to those of the younger elderly, which were lower than those of the elderly in the same age group in developing countries. However what cannot be ignored was that the increasing trend of BP levels for the past 16 years, which was worth paying attention to.

The prevalence of hypertension has gradually increased for the past 16 years was consistent with hypertension prevalence and increasing cardiovascular disease burden. And compared with previous studies based adults or younger elderly, the oldest-old had the highest prevalence [11]. Our study also provides data on ISH, which was an important subtype of serious harm among elderly. Data showed that about half of the hypertension oldest-old were classified as ISH subtype, which was higher than the situation in other age groups [12–14]. The result from our study showed that there was no less than 30% percent of total hypertension patients could be classified as stage II and above. This suggested that we need to pay attention to the serious situation of high ISH prevalence and high percentage of stage II & III hypertension in the oldest-old and prevent subsequent cardiovascular diseases.

In addition, it is worth noting that not only the hypertension prevalence was increasing, the prevalence of high-normal BP also showed an increasing trend. And evidence showed that high-normal BP was one of the important risk factors of hypertension and cardiovascular diseases [15]. Without enough early prevention measures, those who had high-normal BP will sooner or later develop into hypertension.

We also added information about related factors associated with hypertension (including ISH) prevalence. The gender difference was not significant, which was unlike adults and in line with expectations, since the protective effect of estrogen had disappeared for more than 30 years[16–19]. For unhealthy lifestyles, those who were ever smoking or alcohol drinking had higher prevalence. This was in line with the phenomenon of quitting smoking or stop drinking because of disease [20]. Obesity was a risk factor for hypertension, just like other studies about adults. Evidence showed that there were a series of endocrine and metabolic changes due to obesity, which might be associated with the hypertension [21].

There were several strengths. First, the CLHLS study was a large scale nationwide study covering 23 provinces of China for the past 16 years. The large sample was unique for representation of the oldest-old. Second, there was good design and strict quality control during the whole survey, which ensures the good quality data.

Our study had several limitations. First, the study sample was from 23 provinces, there was a lack of representativeness for other unselected provinces. Besides, because of the sampling method used in CLHLS, the participants were not representative samples. We calculated both the crude and weighted prevalence, and the trends were similar. Second, most of the participants of CLHLS were from communities, and there was only less than 5% who lived in living in nursing homes or other institutions. But this was in accordance with the situation in China, since more than 95% of the elderly were home-based care. Third, there was less than 3.5% frail oldest-old with severe diseases or disability who didn’t have BP recorded,, and this may cause underestimation of prevalence. Fourth, BP levels were measured on the same day. Although the average of two times was use, long-time changes were not captured. Fifth, we didn’t have information about treatment and medication information. Although this kind of definition had been adopted in quite a number of epidemiological investigations, the resulting bias cannot be ignored. Combined with the results of previous studies on treatment rates in China and the prevalence of hypertension in this study, this bias may lead to an overestimation. However, taking 2014 survey as an example, there were a total of 299 participants who were classified as hypertension according to previous history with SBP ≤ 140 mmHg and DBP ≤ 90 mmHg this time. And the misclassification participants were calculated as 299–299*30% (the treatment rate according to the China PEACE Million Persons Project) = 209, and the misclassification rate = 209/4587 = 4.6%. this number times. Sixth, only cross-sectional analysis was conducted, and there was lack of the lack of survival data analysis and sensitivity analysis in relation to survival of at least 1–2 years. Seventh, due to the cross-sectional nature, the results about risk factor of prevalence were of low evidence level.

## Conclusions

In conclusion, this was the first large scale nationwide study about hypertension with long time comparisons for over 16 years among oldest-old in Chinese. The results also provided evidence about the trends of hypertension prevalence in China, which indicated that hypertension prevention was still a long and arduous task.

## Data Availability

All data used in this study was stored at http://opendata.pku.edu.cn and available upon request.

## Appendix

**Table 3:** General characteristic of the seven survey waves

Wave	Total population	Total oldest-old	Excluded oldest-old due to missing information	Included population	Mean age	Male (%)
1998	9093	8959	265	8694	92.3 ± 7.6	39.7
2000	11,200	11,162	354	10,808	91.3 ± 7.5	41.6
2002	16,064	11,175	102	11,073	92.6 ± 7.6	39.4
2005	15,638	10,658	160	10,498	92.8 ± 7.2	39.2
2008	16,540	12,002	701	11,301	92.7 ± 7.4	39.4
2011	9765	6530	129	6401	92.2 ± 7.7	40.2
2014	7192	4738	151	4587	91.3 ± 7.6	41.3

**Table 4:** SBP levels by seven waves

Variable	Wave	1998	2000	2002	2005	2008	2011	2014	p
SBP	Gender								
	Male	147.9 ± 23.6	137.5 ± 21.1	134.7 ± 17.1	131.1 ± 18.1	135.5 ± 20.9	136.2 ± 20.4	139.9 ± 20.8	< 0.001
	Female	148.8 ± 25.1	137.6 ± 22.3	134.3 ± 17.5	130.6 ± 19.0	136.1 ± 21.3	137.1 ± 23.0	139.4 ± 22.8	< 0.001
	p	0.078	0.825	0.195	0.064	0.181	0.102	0.100	
	Age-group								
	80–89 yrs	150.9 ± 24.3	138.5 ± 22.3	135.5 ± 18.0	132.4 ± 18.7	138.0 ± 21.8	137.8 ± 20.7	140.4 ± 20.8	< 0.001
	90–99 yrs	148.6 ± 24.0	138.0 ± 22.0	134.5 ± 17.2	131.1 ± 18.7	135.5 ± 21.3	137.5 ± 22.6	139.4 ± 22.4	< 0.001
	100- yrs	144.7 ± 25.1	135.0 ± 20.3	133.1 ± 16.6	127.9 ± 18.2	133.5 ± 19.8	134.5 ± 23.4	137.6 ± 23.9	< 0.001
	p _for trend_	< 0.001	< 0.001	< 0.001	< 0.001	< 0.001	< 0.001	0.007	
	Category of residence								
	City	148.3 ± 24.0	137.2 ± 21.8	134.6 ± 18.3	130.3 ± 18.7	133.8 ± 18.7	133.2 ± 20.2	134.1 ± 20.0	< 0.001
	Town		137.4 ± 21.3	135.1 ± 17.2	130.8 ± 18.4	134.8 ± 20.5	136.7 ± 21.2	138.9 ± 20.6	< 0.001
	Rural	148.6 ± 24.8	138.0 ± 22.1	134.2 ± 17.0	130.9 ± 18.8	136.9 ± 20.1	138.4 ± 23.0	141.2 ± 22.9	< 0.001
	p _for trend_	0.587	0.277	0.087	0.496	< 0.001	< 0.001	< 0.001	
	Region of China								
	East	149.0 ± 24.0	139.0 ± 21.1	134.6 ± 17.1	132.8 ± 21.6	139.7 ± 22.7	139.2 ± 22.3	140.2 ± 22.4	< 0.001
	Central	148.1 ± 24.8	134.8 ± 22.3	134.2 ± 18.4	130.6 ± 17.6	135.3 ± 20.5	134.4 ± 21.8	139.8 ± 22.2	< 0.001
	West	146.6 ± 25.2	133.0 ± 20.9	132.2 ± 16.8	127.9 ± 16.4	135.5 ± 21.9	134.0 ± 20.0	136.7 ± 19.1	< 0.001
	p _for trend_	0.006	< 0.001	< 0.001	< 0.001	< 0.001	< 0.001	0.007	
	Total	148.4 ± 24.4	137.0 ± 21.6	134.1 ± 17.4	130.8 ± 18.7	136.1 ± 21.4	137.1 ± 22.0	139.7 ± 22.0	< 0.001
SBP among those without hypertension	Gender								
	Male	135.7 ± 19.5	126.3 ± 16.6	126.4 ± 14.2	120.4 ± 10.7	122.7 ± 11.2	121.6 ± 11.7	123.3 ± 10.9	< 0.001
	Female	136.0 ± 21.3	125.6 ± 17.4	125.2 ± 13.6	119.1 ± 11.3	122.5 ± 11.1	120.7 ± 12.4	121.9 ± 11.9	< 0.001
	p	< 0.001	0.176	0.002	0.097	0.751	0.981	0.317	
	Age-group								
	80–89 yrs	138.5 ± 20.4	126.7 ± 17.8	126.3 ± 14.2	120.4 ± 10.9	123.2 ± 11.2	122.4 ± 11.5	123.9 ± 10.4	< 0.001
	90–99 yrs	135.7 ± 20.0	126.2 ± 17.2	125.8 ± 13.9	120.3 ± 10.7	122.1 ± 11.3	121.2 ± 12.1	122.5 ± 11.6	< 0.001
	100- yrs	132.8 ± 21.1	123.8 ± 15.3	124.7 ± 13.4	117.7 ± 11.6	122.5 ± 10.9	119.1 ± 12.8	119.6 ± 13.0	< 0.001
	p _for trend_	< 0.001	< 0.001	0.001	< 0.001	0.008	< 0.001	< 0.001	
	Category of residence								
	City	135.6 ± 20.3	125.8 ± 17.1	125.4 ± 13.6	118.9 ± 10.8	122.3 ± 10.6	120.5 ± 12.1	120.7 ± 11.3	< 0.001
	Town		125.9 ± 16.4	125.6 ± 13.5	119.0 ± 11.5	122.6 ± 11.0	120.9 ± 12.3	123.4 ± 11.8	< 0.001
	Rural	136.5 ± 20.8	125.9 ± 17.6	126.3 ± 15.0	120.1 ± 11.1	122.7 ± 11.4	121.8 ± 11.8	122.5 ± 11.4	< 0.001
	p _for trend_	0.142	0.957	0.089	0.001	0.722	0.062	0.004	
	Region of China								
	East	136.5 ± 20.1	127.4 ± 17.3	126.1 ± 13.6	122.4 ± 12.1	123.5 ± 10.5	122.4 ± 11.6	123.4 ± 11.4	< 0.001
	Central	136.4 ± 21.5	123.7 ± 16.9	126.0 ± 15.5	120.1 ± 10.9	122.4 ± 12.1	121.9 ± 11.6	122.6 ± 11.4	< 0.001
	West	133.5 ± 20.6	123.7 ± 14.7	123.7 ± 12.1	118.5 ± 10.5	120.7 ± 11.9	118.8 ± 12.8	121.8 ± 11.5	< 0.001
	p _for trend_	0.001	< 0.001	< 0.001	< 0.001	< 0.001	< 0.001	0.179	
	Total	136.0 ± 20.6	125.7 ± 16.9	119.7 ± 11.1	119.6 ± 11.1	122.6 ± 11.2	121.1 ± 12.1	122.5 ± 11.5	< 0.001

*:City and town were combined as one category in 1998 wave

**Table 5:** DBP levels by seven waves

Variable	Wave	1998	2000	2002	2005	2008	2011	2014	p
DBP	Gender								
	Male	84.5 ± 13.3	81.9 ± 12.8	85.2 ± 12.0	82.5 ± 11.7	79.2 ± 11.9	79.2 ± 11.8	79.8 ± 11.8	< 0.001
	Female	84.4 ± 13.6	81.7 ± 13.0	85.5 ± 12.3	81.8 ± 12.1	78.7 ± 11.4	79.7 ± 12.5	79.4 ± 11.8	< 0.001
	p	0.854	0.419	0.216	0.007	0.031	0.126	0.285	
	Age-group								
	80–89 yrs	85.5 ± 13.3	82.1 ± 13.6	85.5 ± 12.7	83.2 ± 12.2	79.0 ± 11.6	80.2 ± 11.8	80.3 ± 11.4	< 0.001
	90–99 yrs	84.7 ± 13.4	81.9 ± 13.1	85.5 ± 11.9	81.9 ± 11.9	79.0 ± 12.0	79.4 ± 12.2	79.2 ± 12.0	< 0.001
	100- yrs	82.6 ± 13.7	81.0 ± 12.3	85.0 ± 11.8	80.9 ± 11.8	78.6 ± 11.2	78.4 ± 13.4	78.6 ± 12.3	0.854
	p _for trend_	< 0.001	0.003	0.119	< 0.001	0.121	< 0.001	< 0.001	
	Category of residence								
	City	83.6 ± 13.0	81.5 ± 14.0	84.3 ± 12.2	81.7 ± 11.7	79.6 ± 12.5	77.4 ± 12.3	77.3 ± 12.0	< 0.001
	Town		81.7 ± 13.0	85.7 ± 12.2	81.8 ± 12.4	78.8 ± 11.7	80.0 ± 11.9	79.6 ± 11.2	< 0.001
	Rural	84.9 ± 13.7	82.2 ± 11.9	85.8 ± 12.1	83.4 ± 11.9	78.9 ± 11.2	80.2 ± 12.8	80.2 ± 12.0	< 0.001
	p _for trend_	< 0.001	0.044	< 0.001	< 0.001	< 0.001	< 0.001	< 0.001	
	Region of China								
	East	85.7 ± 12.8	82.5 ± 12.9	85.1 ± 11.4	84.0 ± 10.8	79.5 ± 10.9	80.6 ± 11.7	81.0 ± 11.6	< 0.001
	Central	84.1 ± 14.2	82.1 ± 13.4	84.6 ± 12.0	80.1 ± 13.7	79.4 ± 12.9	80.2 ± 12.3	80.2 ± 12.4	< 0.001
	West	82.9 ± 14.4	79.5 ± 12.7	82.6 ± 12.3	79.1 ± 11.0	76.5 ± 11.8	78.6 ± 12.2	79.0 ± 11.4	< 0.001
	p _for trend_	< 0.001	< 0.001	< 0.001	< 0.001	< 0.001	< 0.001	< 0.001	
	Total	84.3 ± 13.4	81.8 ± 13.0	84.6 ± 11.7	82.0 ± 11.9	78.9 ± 11.7	79.4 ± 12.0	79.7 ± 11.8	< 0.001
DBP among those without hypertension	Gender								
	Male	80.3 ± 10.7	78.1 ± 9.6	80.6 ± 8.2	76.6 ± 7.3	75.3 ± 8.7	73.9 ± 8.8	74.5 ± 8.0	< 0.001
	Female	80.0 ± 11.0	77.8 ± 9.5	80.3 ± 8.1	75.6 ± 7.9	75.3 ± 8.6	74.6 ± 9.0	73.7 ± 8.8	< 0.001
	p	0.305	0.184	0.120	< 0.001	0.794	0.323	0.034	
	Age-group								
	80–89 yrs	81.3 ± 10.6	78.1 ± 9.6	80.6 ± 8.1	76.7 ± 7.5	75.8 ± 8.5	74.2 ± 8.4	74.9 ± 7.9	< 0.001
	90–99 yrs	80.3 ± 10.7	78.1 ± 9.4	80.4 ± 8.0	76.1 ± 7.6	75.2 ± 8.5	73.9 ± 8.9	73.7 ± 8.7	< 0.001
	100- yrs	78.4 ± 11.2	77.2 ± 9.7	80.4 ± 8.3	75.1 ± 7.9	75.0 ± 8.8	72.8 ± 9.5	73.0 ± 9.2	0.712
	p _for trend_	< 0.001	0.011	0.652	< 0.001	0.009	0.003	< 0.001	
	Category of residence								
	City	79.9 ± 10.6	77.7 ± 10.0	80.1 ± 8.5	75.5 ± 7.3	75.1 ± 8.9	72.4 ± 9.2	72.9 ± 8.6	< 0.001
	Town		78.0 ± 9.6	80.5 ± 7.8	76.0 ± 7.5	75.5 ± 8.3	73.8 ± 8.3	74.2 ± 8.5	< 0.001
	Rural	80.2 ± 11.0	78.0 ± 9.3	80.7 ± 8.1	76.4 ± 7.9	75.8 ± 8.6	74.5 ± 9.1	74.4 ± 8.4	< 0.001
	p _for trend_	0.321	0.684	0.112	0.019	0.063	< 0.001	0.038	
	Region of China								
	East	81.0 ± 10.5	78.3 ± 9.8	80.9 ± 7.9	77.6 ± 6.5	76.2 ± 8.0	74.5 ± 8.9	74.9 ± 8.6	< 0.001
	Central	80.2 ± 11.2	77.9 ± 9.0	80.7 ± 8.6	75.4 ± 8.7	75.2 ± 9.4	73.8 ± 9.2	74.4 ± 8.6	< 0.001
	West	78.2 ± 11.7	76.5 ± 9.5	78.0 ± 7.4	73.3 ± 7.9	71.7 ± 9.3	73.4 ± 8.4	74.0 ± 7.8	< 0.001
	p _for trend_	< 0.001	< 0.001	< 0.001	< 0.001	< 0.001	0.057	0.265	
	Total	80.0 ± 10.9	77.9 ± 9.6	80.4 ± 8.1	76.0 ± 7.7	75.3 ± 8.7	73.7 ± 8.9	74.2 ± 8.6	< 0.001

*:City and town were combined as one category in 1998 wave

**Table 6:** MAP levels by seven waves

Variable	Wave	1998	2000	2002	2005	2008	2011	2014	p
MAP	Gender								
	Male	105.7 ± 14.9	100.4 ± 13.4	101.7 ± 12.0	98.8 ± 11.8	98.0 ± 12.4	97.9 ± 13.0	99.5 ± 13.0	< 0.001
	Female	105.9 ± 15.5	100.3 ± 13.9	101.7 ± 12.4	98.0 ± 12.6	97.8 ± 12.0	99.2 ± 14.2	99.6 ± 13.5	< 0.001
	p	0.405	0.695	0.835	0.001	0.551	< 0.001	0.784	
	Age-group								
	80–89 yrs	107.3 ± 15.0	100.8 ± 13.9	102.2 ± 12.7	99.6 ± 12.5	98.7 ± 12.5	99.4 ± 13.1	100.3 ± 12.7	< 0.001
	90–99 yrs	106.0 ± 15.1	100.7 ± 13.9	101.8 ± 11.9	98.3 ± 12.1	97.8 ± 12.5	98.8 ± 13.9	99.2 ± 13.5	< 0.001
	100- yrs	103.3 ± 15.7	99.0 ± 12.9	101.0 ± 11.9	96.5 ± 12.0	96.8 ± 11.1	97.2 ± 14.6	98.3 ± 14.3	0.247
	p _for trend_	< 0.001	< 0.001	< 0.001	< 0.001	< 0.001	< 0.001	< 0.001	
	Category of residence								
	City	105.2 ± 14.7	100.2 ± 14.5	101.2 ± 12.3	98.0 ± 12.3	97.5 ± 11.9	96.0 ± 13.3	96.2 ± 12.8	< 0.001
	Town		100.3 ± 13.6	101.8 ± 12.4	98.3 ± 12.7	97.8 ± 12.1	99.0 ± 13.4	99.4 ± 12.4	< 0.001
	Rural	106.1 ± 15.6	100.6 ± 13.1	102.0 ± 12.1	99.1 ± 12.3	98.0 ± 12.3	99.4 ± 14.0	100.5 ± 13.7	< 0.001
	p _for trend_	0.005	0.451	0.034	0.008	0.214	< 0.001	< 0.001	
	Region of China								
	East	106.5 ± 14.7	101.0 ± 13.4	101.6 ± 11.7	99.5 ± 11.5	98.1 ± 11.6	98.8 ± 13.3	100.6 ± 13.1	< 0.001
	Central	105.7 ± 15.8	99.9 ± 14.5	101.1 ± 12.3	97.0 ± 13.8	98.0 ± 13.2	98.5 ± 14.0	99.4 ± 13.9	< 0.001
	West	104.1 ± 16.1	97.3 ± 13.2	99.1 ± 12.0	96.0 ± 11.2	97.6 ± 12.7	98.2 ± 13.6	99.0 ± 12.2	< 0.001
	p _for trend_	< 0.001	< 0.001	< 0.001	< 0.001	< 0.001	0.459	0.020	< 0.001
	Total	105.6 ± 15.2	100.2 ± 13.7	101.1 ± 12.0	98.2 ± 12.2	98.0 ± 12.3	98.6 ± 13.6	99.7 ± 13.3	< 0.001
MAP among those without hypertension	Gender								
	Male	98.8 ± 12.2	94.1 ± 10.5	95.9 ± 8.7	91.2 ± 7.1	91.1 ± 8.0	89.8 ± 8.6	90.7 ± 7.8	< 0.001
	Female	98.7 ± 13.0	93.7 ± 10.6	95.3 ± 8.6	90.1 ± 7.7	91.1 ± 7.8	89.3 ± 8.9	89.8 ± 8.5	< 0.001
	p	0.732	0.107	0.006	< 0.001	0.990	0.106	0.007	
	Age-group								
	80–89 yrs	100.4 ± 12.3	94.3 ± 10.8	95.7 ± 8.7	91.3 ± 7.3	91.0 ± 7.7	90.3 ± 8.2	91.2 ± 7.5	< 0.001
	90–99 yrs	98.8 ± 12.4	94.1 ± 10.5	95.7 ± 8.6	90.8 ± 7.2	90.8 ± 7.9	89.6 ± 8.8	90.0 ± 8.3	< 0.001
	100- yrs	96.5 ± 13.2	92.8 ± 10.2	95.2 ± 8.8	89.3 ± 7.9	91.4 ± 7.9	88.2 ± 9.4	88.5 ± 9.3	< 0.001
	p _for trend_	< 0.001	< 0.001	0.112	< 0.001	0.099	< 0.001	< 0.001	
	Category of residence								
	City	98.7 ± 12.4	94.0 ± 10.9	95.5 ± 9.3	90.0 ± 7.3	90.9 ± 7.8	88.4 ± 8.8	88.9 ± 8.5	< 0.001
	Town		94.0 ± 10.3	95.5 ± 8.4	90.6 ± 7.6	91.2 ± 7.5	89.5 ± 8.3	90.3 ± 8.4	< 0.001
	Rural	98.8 ± 12.9	97.8 ± 10.5	95.7 ± 8.5	90.7 ± 7.5	91.3 ± 8.0	90.3 ± 8.9	90.7 ± 8.1	< 0.001
	p _for trend_	0.821	0.795	0.742	0.011	0.340	< 0.001	0.005	
	Region of China								
	East	99.5 ± 13.1	94.6 ± 10.8	96.0 ± 8.6	91.8 ± 6.6	91.9 ± 7.3	90.3 ± 8.3	91.1 ± 7.7	< 0.001
	Central	98.9 ± 12.3	93.2 ± 10.2	95.8 ± 9.2	90.4 ± 8.0	90.4 ± 8.5	89.7 ± 8.5	90.2 ± 8.3	< 0.001
	West	96.7 ± 13.2	92.2 ± 9.6	93.2 ± 8.0	88.3 ± 8.1	88.6 ± 8.3	88.8 ± 9.2	90.2 ± 8.4	< 0.001
	p _for trend_	< 0.001	< 0.001	< 0.001	< 0.001	< 0.001	< 0.001	0.316	
	Total	98.7 ± 12.7	93.8 ± 10.5	95.5 ± 8.7	90.6 ± 7.5	91.0 ± 7.9	89.6 ± 8.7	90.3 ± 8.3	< 0.001

*:City and town were combined as one category in 1998 wave

**Table 7:** PP levels by seven waves

Variable	Wave	1998	2000	2002	2005	2008	2011	2014	p
PP	Gender								
	Male	63.4 ± 19.1	55.6 ± 18.8	49.6 ± 15.0	48.9 ± 16.5	56.3 ± 19.9	56.0 ± 16.8	59.1 ± 17.6	< 0.001
	Female	64.4 ± 20.3	55.9 ± 19.5	48.8 ± 14.8	48.5 ± 16.5	57.4 ± 20.4	58.4 ± 18.8	60.6 ± 19.4	< 0.001
	p	0.021	0.427	0.012	0.195	0.008	< 0.001	0.009	
	Age-group								
	80–89 yrs	65.4 ± 19.9	56.6 ± 19.6	49.9 ± 15.3	49.2 ± 16.5	59.0 ± 20.3	58.0 ± 17.0	60.2 ± 17.8	< 0.001
	90–99 yrs	63.9 ± 19.6	55.8 ± 19.4	49.0 ± 15.0	49.2 ± 17.0	56.5 ± 20.3	57.7 ± 18.6	60.1 ± 19.3	< 0.001
	100- yrs	62.1 ± 19.9	54.0 ± 18.1	48.1 ± 14.5	47.1 ± 15.7	55.0 ± 20.0	56.0 ± 19.0	59.0 ± 19.8	0.247
	p _for trend_	< 0.001	< 0.001	< 0.001	< 0.001	< 0.001	0.004	0.244	
	Category of residence								
	City	63.6 ± 19.9	55.1 ± 19.0	48.5 ± 14.6	46.9 ± 15.8	54.0 ± 18.9	55.8 ± 16.6	56.3 ± 17.1	< 0.001
	Town		55.5 ± 18.7	48.8 ± 14.2	49.1 ± 15.4	55.8 ± 19.5	56.5 ± 17.0	59.8 ± 17.9	< 0.001
	Rural	64.6 ± 19.8	56.5 ± 19.7	50.8 ± 16.1	49.1 ± 17.2	58.3 ± 20.7	58.5 ± 19.0	61.1 ± 19.4	< 0.001
	p _for trend_	0.024	0.008	< 0.001	< 0.001	< 0.001	< 0.001	< 0.001	
	Region of China								
	East	64.9 ± 19.5	56.9 ± 19.0	49.6 ± 14.4	53.7 ± 20.3	63.2 ± 21.6	60.6 ± 19.0	61.1 ± 19.5	< 0.001
	Central	63.7 ± 20.0	53.5 ± 18.5	49.5 ± 16.1	47.8 ± 14.2	56.0 ± 20.9	53.8 ± 17.0	58.8 ± 18.0	< 0.001
	West	62.4 ± 20.4	52.3 ± 18.6	49.4 ± 15.2	46.7 ± 14.7	55.9 ± 19.5	53.7 ± 14.5	56.5 ± 16.3	< 0.001
	p _for trend_	< 0.001	0.908	< 0.001	< 0.001	< 0.001	< 0.001	< 0.001	< 0.001
	Total	64.1 ± 19.8	55.2 ± 18.9	49.5 ± 15.0	48.8 ± 16.6	57.1 ± 20.5	57.7 ± 18.1	59.9 ± 18.8	< 0.001
PP among those without hypertension	Gender								
	Male	55.4 ± 15.7	48.2 ± 14.1	45.7 ± 12.6	43.8 ± 10.3	47.4 ± 11.3	47.7 ± 10.2	48.8 ± 9.7	< 0.001
	Female	56.0 ± 16.7	47.8 ± 14.8	44.9 ± 11.9	43.4 ± 10.5	47.2 ± 11.7	47.1 ± 10.8	48.2 ± 11.0	< 0.001
	p	0.209	0.377	0.006	0.189	0.543	0.127	0.247	
	Age-group								
	80–89 yrs	57.2 ± 16.2	48.6 ± 15.1	45.9 ± 12.6	44.2 ± 10.4	48.2 ± 11.7	48.1 ± 10.3	49.0 ± 9.8	< 0.001
	90–99 yrs	55.5 ± 16.1	48.1 ± 14.7	45.3 ± 12.3	43.8 ± 10.3	46.9 ± 11.4	47.3 ± 10.4	48.8 ± 10.9	< 0.001
	100- yrs	54.4 ± 16.1	46.6 ± 13.0	44.3 ± 11.5	42.6 ± 10.4	46.7 ± 11.5	46.3 ± 11.0	46.6 ± 11.0	0.611
	p _for trend_	< 0.001	< 0.001	< 0.001	< 0.001	< 0.001	< 0.001	< 0.001	
	Category of residence								
	City	55.5 ± 16.3	47.9 ± 14.3	44.9 ± 12.0	42.6 ± 9.6	46.6 ± 11.4	47.1 ± 11.0	47.8 ± 10.1	< 0.001
	Town		48.0 ± 14.1	45.0 ± 11.8	43.3 ± 10.5	47.2 ± 11.6	47.4 ± 10.0	48.3 ± 10.5	< 0.001
	Rural	56.3 ± 16.4	48.0 ± 15.1	46.3 ± 12.8	44.0 ± 10.6	47.5 ± 11.6	48.1 ± 10.7	49.0 ± 10.6	< 0.001
	p _for trend_	0.012	0.960	0.001	< 0.001	0.059	0.150	0.210	
	Region of China								
	East	56.3 ± 15.9	49.1 ± 14.6	45.7 ± 11.4	42.4 ± 10.2	47.3 ± 10.7	49.0 ± 10.6	48.7 ± 10.4	< 0.001
	Central	55.5 ± 17.3	45.7 ± 14.2	45.3 ± 14.3	45.2 ± 10.0	45.5 ± 12.3	44.9 ± 10.6	48.4 ± 10.2	< 0.001
	West	55.3 ± 16.1	47.3 ± 13.5	45.2 ± 11.3	44.8 ± 11.0	50.7 ± 13.1	47.4 ± 9.9	47.4 ± 10.6	< 0.001
	p _for trend_	0.190	< 0.001	0.514	< 0.001	< 0.001	< 0.001	0.078	
	Total	55.9 ± 16.3	47.8 ± 14.4	45.3 ± 12.2	43.6 ± 10.4	47.2 ± 11.7	47.5 ± 10.6	48.3 ± 10.4	< 0.001

*:City and town were combined as one category in 1998 wave

**Table 8:** The prevalence (%) of ISH by seven waves

Wave	1998	2000	2002	2005	2008	2011	2014	p
Gender								
Male	30.6(29.0–32.1)	24.8(23.6–26.1)	15.3(14.2–16.3)	15.0(13.9–16.1)	26.5(25.2–27.8)	50.1(48.1–52.1)	29.0(26.9–31.0)	< 0.001
Female	30.5(29.2–31.7)	25.2(24.1–26.3)	13.7(12.9–14.6)	13.8(13.0–14.7)	28.2(27.1–29.3)	23.5(21.9–25.2)	31.9(30.1–33.6)	< 0.001
p	0.930	0.669	0.025	0.100	0.054	< 0.001	0.036	
Age-group								
80–89 yrs	31.3(29.7–32.8)	25.1(23.9–26.3)	15.1(14.0–16.1)	15.6(14.4–16.7)	30.4(29.0–31.8)	26.2(24.5–27.9)	30.8(28.832.7)	< 0.001
90–99 yrs	30.1(28.5–31.8)	25.6(24.2–27.0)	14.2(13.1–15.3)	14.8(13.7–15.9)	24.0(26.0–28.7)	27.1(25.4–28.9)	30.5(28.2–32.7)	< 0.001
100- yrs	30.0(28.1–31.8)	24.1(22.3–25.8)	13.5(12.3–14.7)	11.7(10.5–12.9)	23.4(22.4–25.5)	23.3(21.0–25.5)	30.7(27.6–33.9)	< 0.001
p _for trend_	0.269	0.491	0.043	< 0.001	< 0.001	0.098	0.941	
Category of residence								
City	29.1(27.9–30.3)	22.8(21.4–24.3)	13.5(12.7–14.4)	11.6(10.3–13.0)	23.0(21.3–24.7)	22.4(19.9–24.9)	26.9(23.4–30.4)	< 0.001
Town		25.0(23.5–26.4)	14.2(12.9–15.6)	14.6(13.7–15.5)	26.2(24.4–28.1)	24.2(22.3–26.1)	31.4(29.6–33.2)	< 0.001
Rural	33.0(31.4–34.6)	26.9(25.6–28.3)	16.2(14.8–17.6)	15.8(14.4–17.2)	29.5(28.4–30.6)	28.0(26.5–29.5)	30.9(28.5–33.3)	< 0.001
p _for trend_	< 0.001	< 0.001	0.005	< 0.001	< 0.001	< 0.001	0.084	
Region of China								
East	32.4(31.3–33.8)	27.1(25.9–28.2)	16.1(14.7–17.5)	21.1(19.5–22.6)	31.0(29.9–32.1)	31.7(30.1–33.2)	32.4(30.5–34.2)	< 0.001
Central	30.3(27.9–32.8)	19.4(17.9–20.9)	14.9(14.0–15.8)	14.1(12.4–15.7)	27.9(26.3–29.4)	20.7(18.8–22.6)	28.7(26.2–31.1)	< 0.001
West	27.5(25.5–29.6)	19.2(17.2–21.2)	14.1(12.4–15.8)	11.4(10.4–12.2)	20.3(18.0–22.6)	17.0(14.5–19.6)	26.3(22.4–30.2)	< 0.001
p _for trend_	< 0.001	< 0.001	0.153	< 0.001	< 0.001	< 0.001	0.002	
Total	30.5(29.6–31.5)	25.1(24.2–25.9)	14.3(13.7–15.0)	14.3(13.6–14.9)	27.5(26.7–28.4)	25.9(24.8–27.0)	30.7(29.3–32.0)	< 0.001
Weighted Total^†^	30.5(29.5–31.4)	26.6(25.8–27.4)	14.0(12.7–15.4)	14.8(13.4–16.2)	30.2(28.4–32.0)	23.7(21.9–25.6)	30.1(27.9–32.2)	< 0.001
Weighted Total^†^	31.2(30.2–32.1)	25.2(24.4–26.0)	14.9(14.2–15.6)	15.4(14.7–16.1)	30.1(29.3–31.0)	26.5(25.4–27.5)	30.9(29.6–32.2)	< 0.001

*:City and town were combined as one category in 1998 wave

^†^: Weight was calculated based on age-sex-residence-specific distribution from the CLHLS study

^‡^: Weight was calculated based on the sixth national census data

**Table 9:** BP levels by seven waves among those who were first included in each survey wave

Variable	1998	2000	2002	2005	2008	2011	2014	p
mean ± SD								
SBP	148.5 ± 24.5	137.6 ± 21.8	133.3 ± 16.3	130.6 ± 18.7	136.3 ± 21.0	139.6 ± 22.6	142.2 ± 22.2	< 0.001
DBP	84.4 ± 13.5	81.8 ± 12.9	85.4 ± 11.7	82.1 ± 12.1	79.0 ± 11.3	79.7 ± 11.8	79.6 ± 12.2	< 0.001
MAP	64.0 ± 19.9	55.8 ± 19.2	47.9 ± 13.9	48.6 ± 16.4	57.4 ± 20.2	59.9 ± 19.0	62.6 ± 18.7	< 0.001
PP	105.8 ± 15.3	100.4 ± 13.7	101.4 ± 11.6	98.2 ± 12.3	98.1 ± 11.9	99.7 ± 13.5	100.5 ± 13.6	< 0.001
%								
Prevalence	43.1(42.0–44.1)	43.6(42.7–44.6)	41.2(39.4–42.9)	47.1(46.1–48.1)	46.4(45.3–47.6)	58.8(56.5–61.0)	62.3(59.7–64.9)	< 0.001

## Abbreviations

BP: Blood pressure
CI: Confidence interval
CLHLS: Chinese Longitudinal Healthy Longevity Survey
DBP: Diastolic blood pressure
MAP: Mean arterial pressure
NHANES: National Health and Nutrition Examination Survey
OR: Odds ratio
PP: Pulse pressure
SBP: Systolic blood pressure
